# Recombinant Dense Granule Protein (NcGRA4) Is a Novel Serological Marker for *Neospora caninum* Infection in Goats

**DOI:** 10.3390/ani13111879

**Published:** 2023-06-05

**Authors:** Ruenruetai Udonsom, Aongart Mahittikorn, Apichai Prachasuphap, Kodcharad Jongpitisub, Panadda Dhepakson, Charoonluk Jirapattharasate

**Affiliations:** 1 Department of Protozoology, Faculty of Tropical Medicine, Mahidol University, 420/6 Ratchawithi Road, Ratchathewi, Bangkok 10400, Thailand; ruenruetai.udo@mahidol.ac.th (R.U.); aongart.mah@mahidol.edu (A.M.); 2 Department of Medical Sciences, Medical Life Sciences Institute, 88/7 Tiwanon Road, Talad Kwan Subdistrict, Muang District, Nonthaburi 11000, Thailand; apichai.p@dmsc.mail.go.th (A.P.); kodcharad.j@dmsc.mail.go.th (K.J.); panadda.d@dmsc.mail.go.th (P.D.); 3 Department of Pre-Clinic and Applied Animal Science, Faculty of Veterinary Science, Mahidol University, 999 Phuthamonthon Sai 4 Rd, Salaya, Nakhon Pathom 73170, Thailand

**Keywords:** recombinant protein, dense granules, serology, *Neospora caninum*, goats

## Abstract

**Simple Summary:**

Neosporosis caused by *Neospora caninum* is now recognised globally as one of the major causes of productivity losses in small and large ruminants. Its diagnosis is primarily based on serological techniques, and various serological tests have been developed utilising native-soluble antigens or live tachyzoites. However, these tests frequently cross-react with proteins from other protozoa. To overcome this problem, we produced a recombinant protein from a synthetic NcGRA4 gene and examined the ability to detect *N. caninum* infection in field samples using indirect enzyme-linked immunosorbent assay (iELISA). The technique demonstrated good specificity and sensitivity, and its use on serum samples from the field revealed that it may be used to effectively diagnose neosporosis in goats.

**Abstract:**

*Neospora caninum* is widely recognised as one of the most significant causes of abortion in cattle, with infections also occurring in sheep and goats. To prevent and control animal neosporosis, it is crucial to develop sensitive and specific methods for detecting *N. caninum* infection. Recently, several recombinant proteins have been utilised in serological assays for the diagnosis of neosporosis. In this study, we used commercial gene synthesis to produce dense granular antigen 4 (NcGRA4) recombinant protein. NcGRA4 plasmids were expressed in the *Escherichia coli* system and then purified. The purified recombinant protein was analysed using sodium dodecyl sulphate–polyacrylamide gel electrophoresis. To evaluate the diagnostic potential of recombinant NcGRA4 protein, we tested 214 serum samples from goat farms via indirect enzyme-linked immunosorbent assay (iELISA) and compared the results to those from the indirect fluorescent antibody test (IFAT). Western blotting analysis revealed a single NcGRA4 band with an expected molecular weight of 32 kDa. The specific IgG against *N. caninum* was detected in 34.1% and 35% of samples evaluated by NcGRA4 iELISA and IFAT, respectively. The sensitivity and specificity of the NcGRA4 iELISA were 71.6% and 86.3%, respectively, when compared with the results from IFAT. Our results demonstrate that a recombinant protein that can be used to detect animal neosporosis can be produced using a synthetic NcGRA4 gene. Overall, recombinant NcGRA4 shows promise as a sensitive and specific serological marker for identifying target IgG in goat samples.

## 1. Introduction

*Neospora caninum* is an obligate intracellular protozoan parasite that is one of the most common infectious causes of abortion in cattle worldwide, leading to significant economic losses for the livestock industry [1]. Both domestic and wild canines are considered definitive hosts for the protozoan, whereas many different types of animals, especially cattle goats and sheep, serve as intermediate hosts [1,2]. While its importance in cattle is widely recognized, this parasite may also potentially pose a significant threat as an abortifacient for small ruminants, and it is possibly the primary cause of reproductive failure for some flocks [2,3,4,5].

Neosporosis-related abortions can be definitively diagnosed when the parasite is found in the tissues of the aborted fetuses [6]. However, the low titre of parasites and the potential for severe autolysis in aborted fetuses means that this is frequently not practicable. Serological detection utilizing various antibodies (Immunoglobulins G and M) is commonly employed to diagnose *N. caninum* infection in various animals. Several serological tests, such as the indirect fluorescence antibody test (IFAT), enzyme-linked immunosorbent assay (ELISA) and Western blot, have been used to assess infection status by detecting anti-*N. caninum* antibodies in animal sera [7,8,9]. The *N. caninum* tachyzoite is commonly used as an antigen [6,8]; however, because *N. caninum* tachyzoites share similar antigens with the closely related protozoan *Toxoplasma gondii*, the use of complete tachyzoites for immunodiagnosis may result in false positives due to cross-reaction [10].

Rhoptries, micronemes and dense granules are specialised *N. caninum* secretory organelles that secrete proteins that are crucial to this protozoan’s intracellular parasitism [11,12]. For example, the *N. caninum* dense granule antigen (NcGRA) is a key component of both the vacuoles enclosing tachyzoites and the cyst wall containing slower-growing bradyzoites, suggesting that NcGRA is a crucial protective antigen that has the potential to be used as a diagnostic tool [13]. Several GRAs have been identified in *N. caninum*, including NcGRA2 [14], NcGRA6 [15,16,17], NcGRA7 [15,17,18], NcGRA9 [19] and NcGRA14 [20]. The potentials of NcGRA6, NcGRA7 and NcGRA14 for the diagnosis of bovine neosporosis have been demonstrated [17,18]. Recently, *N. caninum* GRA4 (designated NcGRA4) was shown to perform well at improving immune protection activity and its use for the serodiagnosis of *N. caninum* infection was suggested [21].

Recombinant *N. caninum* antigens were recently found as promising candidates for replacing native antigens due to due to their simplicity of production in large quantities utilizing standardized methods [22]. A previous study used a synthetic gene to produce a recombinant protein of *T. gondii* [23]. Therefore, here, we designed a synthetic NcGRA4 gene and the resulting recombinant protein was expressed using a bacterial system. The serological diagnostic performance of recombinant NcGRA4 protein was evaluated in a specific IgG indirect ELISA (iELISA) to diagnose *N. caninum* infection in goat sera. The IFAT was employed to validate the detection method developed in this study.

## 2. Materials and Methods

### 2.1. Bioinformatics Analysis of NcGRA4

Protein BLAST (BLASTp) was employed to blast homologies between the amino acid sequences of GRA4 of *N. caninum* and *T. gondii*, which were obtained from an online database (http://ToxoDB.org, accessed on 28 June 2022). The accession numbers of NcGRA4 and TgGRA4 are NCLIV_054830 and TGME49_310780, respectively. Online programmes, including SignalP-5.0 (https://services.healthtech.dtu.dk/service/SignalP-5.0/, accessed on 28 June 2022) and TMHMM-2.0 (https://services.healthtech.dtu.dk/services/TMHMM-2.0/, accessed on 28 June 2022) were used to predict the signal peptides (SPs), cleavage sites and potential transmembrane regions (TMs). 

### 2.2. Gene Synthesis of the NcGRA4 Plasmid

**Figure 1:**
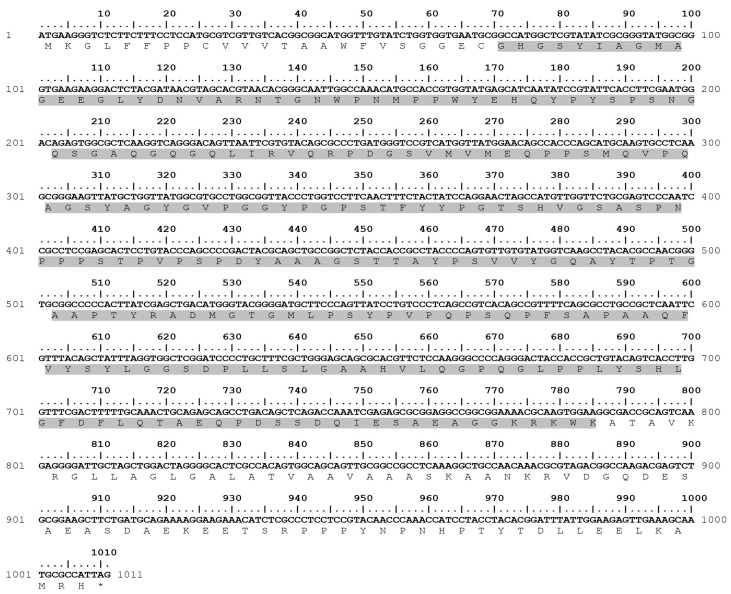
Full nucleotide and amino acid sequences of *N. caninum* GRA4 (accession number: NCLIV_054830). The GRA4 expression area is highlighted by shading (* encoding a 240-residual peptide).

The NcGRA4 sequence is composed of 1011 nucleotides, which code for a 336 amino acid protein. SignalP-5.0 analysis revealed an expressed signal sequence consisting of small fragments at amino acids 1–23. The result of TMs analysis shows that amino acids 264–291 encode for transmembrane regions. Therefore, the encoding amino acids 24–263 were generated (Figure 1) and inserted into a pET-21d vector harbouring an N-terminal FLAG tag (DYKDDDDK) using the cloning sites NcoI and XhoI (General Biosystems, Durham, NC, USA).

### 2.3. Expression of the Recombinant NcGRA4 Protein

The recombinant NcGRA4 plasmid (10 ng/µL) was transformed into *Escherichia coli* BL21 star (DE3) cells (New England Biolabs, Ipswich. MA, USA). The bacteria were cultivated in 2XTY supplemented with 1% glucose and 200 ng/mL ampicillin at 37 °C with shaking at 250 rpm until an optical density (OD) at 600 nm of 0.5 was reached. Protein expression was induced by isopropyl-b-D-thiogalactopyranoside (IPTG) (Thermo Fisher Scientific, Agawam, MA, USA) to a final concentration of 1 mM at various temperatures (20 °C, 25 °C and 30 °C) overnight with shaking at 250 rpm. The induced bacteria were collected through centrifugation at 4400× *g* for 20 min at 4 °C and then the bacterial pellet was weighed. The cell pellet was lysed using the Buster protein extraction reagent (Novagen, Darmstadt, Germany) according to the manufacturer’s instructions. The recombinant NcGRA4 protein was examined via 12% sodium dodecyl sulphate–polyacrylamide gel electrophoresis (SDS-PAGE) and stained with Coomassie blue (Thermo Fisher Scientific, USA). 

### 2.4. Purification of the Recombinant NcGRA4 Protein

The purified protein was obtained using anti-DYKDDDDK agarose beads (Smart Lifesciences, Hong Kong, China). First, the protein homogenate was centrifuged at 10,000× *g* for 30 min at 4 °C and the supernatant was collected in a clean tube. The supernatant was transferred to a 2-mL centrifuge column (Thermo Fisher Scientific, USA) and mixed with anti-DYKDDDDK beads for 5 min at room temperature. The beads were washed three times with Tris-buffered saline (TBS, 50 mM Tris-HCl, 150 mM NaCl, pH 7.4) and then resuspended in 500 µL 0.3 M glycine elution buffer (pH 3.0) and incubated for 5 min. The solution was eluted into a 1.5-mL tube and 10 µL 1 M Tris-HCl, pH 9 was added to neutralise the elution buffer. The protein concentration was determined using a NanoDrop ND-1000 UV/Vis spectrophotometer (Thermo Fisher Scientific, USA). 

The eluted NcGRA4 fractions were pooled and dialysed using SnakeSkin Dialysis Tubing (10 kDa cut-off, Thermo Fisher Scientific, USA) against phosphate-buffered saline (PBS, pH 7.4) at 4 °C. The purified recombinant protein concentration was analysed using both SDS-PAGE and a BCA protein assay kit from Pierce Biotechnology (Rockford, IL, USA) before storage at −20 °C until further use. Furthermore, the amino acid sequence of the NcGRA4 recombinant protein was confirmed by mass spectrometry (MS) analysis performed by the Proteomics Services Centre, Faculty of Medical Technology, Mahidol University. 

### 2.5. Western Blot Analysis

Purified NcGRA4 recombinant proteins (5 µg) were separated by 12% SDS-PAGE and transferred onto a nitrocellulose membrane (Bio-Rad, Hercules, CA, USA). The membrane was washed three times with TBS and incubated with blocking solution (3% non-fat skim milk, Merck, Germany in TBS) for 1 h at 37 °C with constant shaking. Subsequently, the membrane was washed three times with PBS containing 0.01% Tween 20 (PBS-T), followed by a final rinse with PBS. The NcGRA4 recombinant protein in the membrane was incubated at 37 °C for 1 h while shaking constantly with antibody or known reference positive and negative goat sera (diluted 1:200 in 3% skim milk) maintained in our laboratory. The monoclonal antibody (mAb) against FLAG tag (GenScript, Piscataway, NJ, USA) was diluted 1:1000 in blocking buffer, while the polyclonal rabbit anti-goat IgG (H + L) secondary antibody (Thermo Fisher Scientific, USA) was diluted 1:10,000. The membrane was rinsed three times with PBS-T after incubation. The antigen–antibody reaction was detected based on peroxidase activity using 3,3′,5,5′-tetramethylbenzidine (Thermo Fisher Scientific, USA).

### 2.6. Animal Sample

Serum samples were collected as part of a project to test *T. gondii*- and *N. caninum*-specific recombinant proteins for the diagnosis of toxoplasmosis and neosporosis, respectively, in cattle and goats. The Ethics and Animal Care and Use Committee of the Faculty of Veterinary Science, Mahidol University, approved the use of goat sera in this study (Permit Number: MUVS-2023-02-10). A total of 214 serum samples were collected from goat farms in Khon Kaen and Chaiyaphum provinces, Thailand. The goats were restrained by holding the base of the horn, then blood was collected from the jugular vein and immediately transferred into 10-mL vacuum blood tubes without anticoagulant. All blood samples were sent to the laboratory at the Mahidol University Faculty of Tropical Medicine in chilled boxes with ice packs. Following the sedimentation of blood cells, the sera were separated and kept at −20 °C until further examination.

### 2.7. IFAT

Goat IgG antibodies to *N. caninum* were raised using tachyzoites of the Nc1 strain as the antigen. A cut-off value of 1:100 [24] was used with dilutions of caprine sera in FA serum diluting buffer (pH 7.2, VMRD, Pullman, WA, USA). The diluted sera (20 µL) were inoculated into each slide well, then incubated at 37 °C for 1 h in a humid chamber. After incubation, the slides were rinsed and soaked in 1 × FA rinse buffer (VMRD, USA) for 10 min. Rabbit anti-goat IgG solution conjugated with fluorescein isothiocyanate (SeraCare, Milford, MA, USA) was added to each well in a 1:200 dilution in the diluting buffer and then incubated at 37 °C for 1 h in a humid chamber. The slides were then washed as described above and then dried for 10 min. Slides were then mounted with a coverslip using a glycerine solution and observed under a fluorescence microscope (ZEISS Axio Imager 2, ZEISS, Oberkochen, Germany). Positive and negative controls were included in all the slides examined. 

### 2.8. IgG iELISA

The optimal conditions for recombinant NcGRA4 protein-based indirect ELISA were determined by testing the concentration of the NcGRA4 protein and the dilution of serum samples. iELISA was performed in duplicate for all samples. Each well of an ELISA microplate (Nunc, Denmark) was coated with 100 μL of recombinant NcGRA4 protein at a final concentration of 1 and 2 μg/mL in 1× ELISA coating buffer (Bio-Rad, Hercules, CA, USA) at 4 °C overnight. The plates were washed five times with PBS-T before being blocked for 1 h at 37 °C with 5% PBS-skimmed milk (PBS-SM). The blocking solution was washed with PBS-T, then 50 µL of duplicate serum samples diluted to 1:100 and 1:200 in PBS-SM was added to each well. The plates were rinsed five times with PBS-T before adding 50 µL of 1:5000 diluted (in PBS) horseradish-peroxidase-conjugated anti-goat IgG antibodies (Invitrogen, Carlsbad, CA, USA). The plates were incubated for 1 h at 37 °C. After incubation, the plates were rinsed five times with PBS-T before colour development using 3,3′,5,5′-tetramethylbenzidine (Invitrogen, USA). The reaction was stopped after 15 min by adding 50 µL of 0.1 M HCl. A spectrophotometer (Multiskan RC, Thermo Labsystems) was used to read the ELISA plates at 450 nm (OD 450).

### 2.9. Statistical Analyses

In this study, we designated the IFAT as the standard serological test. The results of iELISA and IFAT were compared by their kappa coefficient (k), sensitivity (Se), specificity (Sp), positive predictive value (PPV) and negative predictive value (NPV) with 95% confidence intervals using online software (http://vassarstats.net, accessed on 28 June 2022). The k-values were interpreted as follows: fair (κ = 0.21–0.40), moderate (κ = 0.41–0.60) and substantial (κ = 0.61–0.80).

## 3. Results

### 3.1. Bioinformatics Analysis of NcGRA4 and TgGRA4

**Figure 2:**
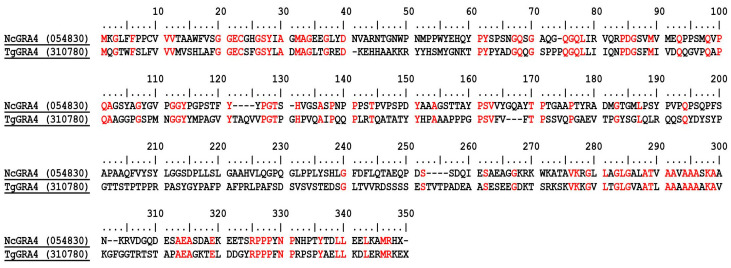
Bioinformatic analysis showing the homology between NcGRA4 (NCLIV_054830) and TgGRA4 (TGME49_310780).

The amino acid sequences of NcGRA4 and TgGRA4 were aligned using Bioedit version 7.2.6 (Tom Hall Ibis Biosciences, Carlsbad, CA, USA). The results of GenBank BLASTp analysis reveal that the proteins share 31.32% similarity (Figure 2).

### 3.2. Expression and Characterisation of Recombinant NcGRA4

**Figure 3:**
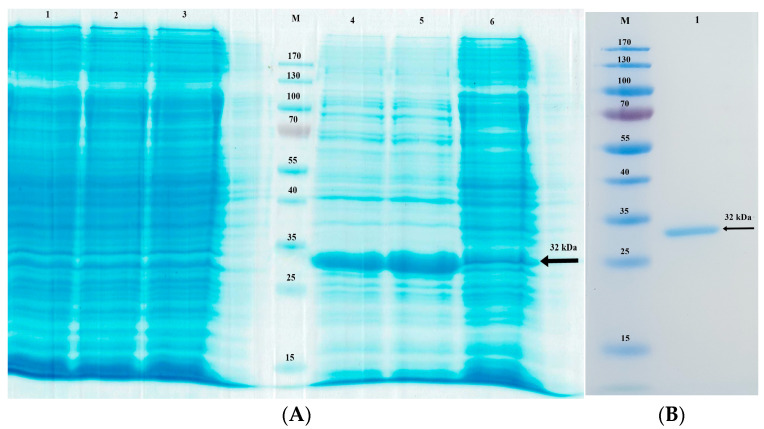
((**A**), Figure S1): Sodium dodecyl sulphate–polyacrylamide gel electrophoresis analysis of the optimal expression of NcGRA4 in E. coli BL21 star (DE3). The gel was stained with Coomassie blue. Lane M: protein molecular weight marker; Lanes 1–3: uninduced culture of *E. coli* BL21 star (DE3)- pET-21d -NcGRA4 (20 °C, 25 °C and 30 °C); Lanes 4–6: pellet fractions of cells induced with 1.0 mM IPTG and cultured at 20 °C, 25 °C and 30 °C. ((**B**), Figure S2) Analysis of the expression of the recombinant NcGRA4 protein by sodium dodecyl sulphate–polyacrylamide gel electrophoresis. Lane M: protein molecular weight marker Lane 1: anti-FLAG tag affinity resin was used to purify the soluble recombinant NcGRA4 protein.

The synthetic NcGRA4 gene fragment cloned in the pET-21d vector that was used to transform *E*. *coli* BL21 star (DE3) cells encodes amino acids 24–263. We optimised the bacterial production of NcGRA4 by expressing the protein at various temperatures and then analysing the products by SDS-PAGE. A 32-kDa band corresponding to NcGRA4 was observed in the induced bacteria, with similar expression levels at 20 °C and 25 °C, as shown in Figure 3A. We validated protein expression in the induced bacteria by purifying the 32-kDa protein band with anti-FLAG tag affinity resin (Figure 3B).

The purified recombinant protein was characterised using MS. The partial sequence of NcGRA4 in the recombinant protein is 100% identical to database sequences (XP_003885086). Therefore, we were able to use MS to confirm that our produced recombinant protein was *N. caninum* GRA4.

### 3.3. Western Blot Analysis of NcGRA4

**Figure 4:**
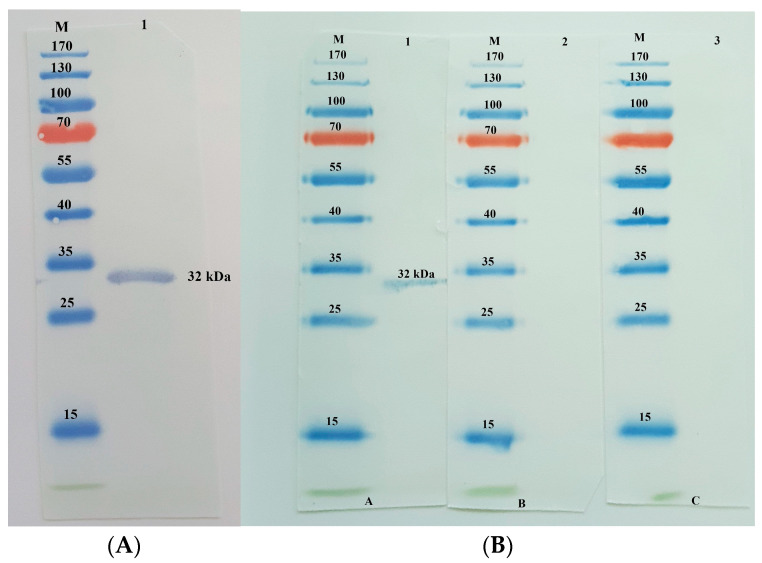
((**A**), Figure S3): Purified recombinant NcGRA4 analysis by Western blotting. Lane M: protein molecular weight marker, Lane 1: the purified 32-kDa NcGRA4 recombinant protein was identified using an anti-FLAG tag antibody ((**B**), Figure S4): Immunoblot analysis, Electrophoresis on 12% sodium dodecyl sulphate–polyacrylamide gels were used to separate the purified proteins, which were then transferred to a nitrocellulose membrane and probed with known positive for *N. caninum* (A), *T. gondii* (B) and negative goat sera (C). Lane M: protein molecular weight marker, Lane 1: strong reactivity with known positive serum, Lanes 2 and 3: results for *T. gondii*-positive serum and *N. caninum*-negative serum.

Western blotting on the soluble fraction protein tagged with a peroxidase-conjugated anti-FLAG tag antibody (GenScript, Piscataway, NJ, USA) diluted 1:1000 in a blocking buffer shows the anti-FLAG tag antibody specifically binding to the NcGRA4 fusion protein (Figure 4A). The specific reactivity and purity of NcGRA4 were tested using known positive samples of *N. caninum* and *T. gondii*, as well as negative serum samples from goats. Western blotting results demonstrate a match between the NcGRA4 fusion protein and the known positive serum. In contrast, no specific band was detected on the blot of the fusion protein probed with negative and positive *T. gondii* serum (Figure 4B).

### 3.4. Evaluation of the Diagnostic Performance of NcGRA4 by iELISA

**Figure 5:**
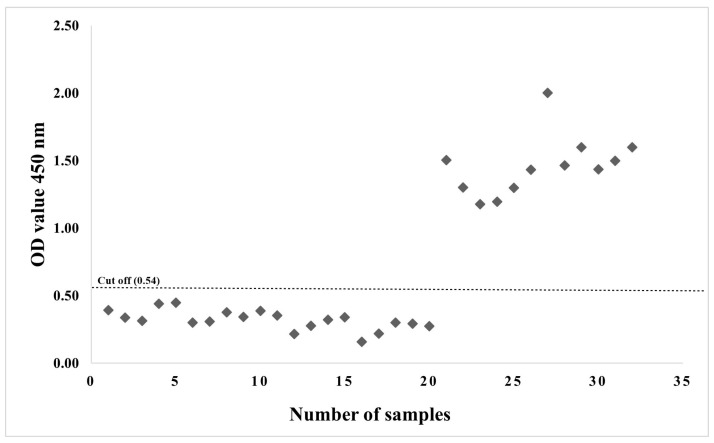
*N. caninum* antibody response to goat field sera utilising recombinant NcGRA4-based indirect enzyme-linked immunosorbent assays. The dashed line represents the cut-off index value (OD450 = 0.54).

The optimal concentration of the purified NcGRA4 antigen was determined using concentrations of 1 and 2 µg/mL along with serum dilutions of 1:100 and 1:200, as shown in Table 1. The twenty negative and twelve positive sera of *N. caninum* infection were confirmed using IFAT and ID Screen^®^ *Neospora caninum* indirect (IDvet, Guichainville, France). The results demonstrated that the optical density (OD) of positive sera increased as the antigen concentration and serum dilution increased, whereas the OD of negative sera was unaffected (Table S1). As determined by the checkerboard assay, the pure NcGRA4 protein concentration of 2 µg/mL and the sample dilution of 1:200 were employed because they provided the most acceptable OD for the tested positive (mean OD = 1.459) and negative (mean OD = 0.320) sera. The serodiagnostic efficacy of recombinant NcGRA4-iELISA was assessed using goat sera. The cut-off value for positive samples was calculated by adding three standard deviations to the average OD_450_ values of the negative controls, yielding a value of 0.54 (Figure 5). The seroprevalence of neosporosis in field serum samples was 34.1% (73/214), based on recombinant NcGRA4-iELISA.

### 3.5. Comparison of iELISA and IFAT

We evaluated the sensitivity, specificity, kappa value, PPV, NPV and agreement proportion of recombinant NcGRA4-iELISA and compared them to those of IFAT. The seroprevalence of anti-IgG *N. caninum* antibodies based on IFAT was 35% (75/214). The level of identity between iELISA and IFAT was moderate, with a kappa (k) coefficient of 0.58. The sensitivity, specificity, PPV and NPV were 71.62%, 86.33%, 73.61% and 85.10 %, respectively (Table 2).

## 4. Discussion

Neosporosis is highly prevalent worldwide and abortions caused by this disease pose a significant problem for livestock farms. Because there is presently no treatment or effective vaccine against neosporosis, early diagnosis and protection are crucial for ruminant farms. Numerous recombinant proteins, e.g., rhoptry proteins, microneme proteins, surface antigens and GRAs [17,18,25,26,27], have been developed and utilised for the diagnosis of *N. caninum* infection. Previous studies have identified surface antigen 1 (SAG1), SRS2-related sequence (SRS2), as the most used antigen for diagnosing *N. caninum* infection in cattle or canines [17,18,22]. The anti-NcSAG1 antibodies have been detected in both acute and chronic *N. caninum* infections [28]. A novel microneme protein 26 (NcMIC26) has recently been identified as an effective microneme protein detected by the sera of *N. caninum*-infected cattle [27]. Among the GRAs proteins, NcGRA6 and NcGRA7 have been identified utilizing various immunodiagnostic methods. Furthermore, anti-NcGRA7 antibodies are usually considered as indicators of acute *N. caninum* infection [29,30]. Recently, during the development of a DNA vaccine for *N. caninum*, workers demonstrated that the recombinant NcGRA4 provides high immune protective efficacy and can be used as an antigen for the development of a safe and effective DNA vaccine against *N. caninum* [21]. To the best of our knowledge, this is the first report on the development of a recombinant NcGRA4 protein that can be used for detecting anti-*N. caninum* antibodies in goats via iELISA. The result of the bioinformatics analysis of NcGRA4 and TgGRA4 reveals that NcGRA4 and TgGRA4 share 31.32% similarity. By using immunoblotting to examine cross-reactivity of *N. caninum* with *T. gondii* using known positive animal sera, we showed that anti-*T. gondii* goat sera do not cross-react with NcGRA4. This finding implies that NcGRA4 is a specific antigen of *N. caninum*, with no cross-reactivity to *T. gondii*. As a result, the NcGRA4 may be utilized to differentiate between these two infectious diseases.

In this study, the gene fragment encoding amino acids 24–263 was used to generate the protein minus the transmembrane region, which formed a distinct 32-kDa band on SDS-PAGE. In the current investigation, however, we noted a size difference between the observed band and the predicted molecular weight of NcGRA4 (26.4 kDa). In *T. gondii*, the dense granule proteins such as GRA3 [31], GRA4 [32], GRA6 [33], GRA7 [34] and GRA8 [35] contain high numbers of proline residues, which confer structural rigidity in the main sequence, thus reducing electrophoretic mobility. Currently, there are no studies on the functions of the NcGRA4 protein. Therefore, the molecular characterisation and constituents of these proteins should be investigated. 

Recombinant DNA technology and synthetic DNA have recently played key roles in the production of high-quality recombinant antigenic proteins for the serological diagnosis of parasitic infection. Furthermore, gene synthesis facilitates the use of the genetic code’s degeneracy to increase the expression of recombinant protein targets for structural research [36]. Moreover, gene synthesis techniques do not require access to a pathogen, saving research personnel from being exposed to potentially hazardous living parasites [37]. Our previous study demonstrated that the recombinant protein produced from a synthetic *T. gondii* GRA8 can be used for an immunodiagnostic test for *T. gondii* infection in goats [23]. Therefore, synthetic genes offer new opportunities to design and construct genes in the absence of an existing DNA template. 

The kappa value (κ = 0.58) between IFAT and NcGRA4-iELISA reveals a moderate agreement. This finding could be implied by the small number of goat samples, resulting in an accurate analysis of the recombinant antigens being difficult. However, the sensitivity and specificity values of recombinant NcGRA4 suggests that it can be employed as an antigen in *N. caninum* serological assays. Recently, a mixture of recombinant antigens (or chimeric antigens) was developed and used as an alternative method for improving the sensitivity and specificity of the diagnostic test for neosporosis [38]. Therefore, the use of NcGRA4 recombinant protein, as well as other recombinant proteins, for the detection of *N. caninum* infection in animals via ELISA should be studied further.

The seroprevalence of *N. caninum* infection in various animals in Thailand has been reported [39,40,41,42,43,44]. However, information and epidemiological data on neosporosis in small ruminants are typically limited in Thailand. A high seroprevalence of *N. caninum* infection was observed in Thai cattle, which ranged from 5.9 to 34.3% [39,40,41,42]. The prevalence of *N. caninum* infection in goat sera in this study (34.1%) is higher than that found in a previous study that was performed in Kanchanaburi province (16.7%) [45], which indicates that *N. caninum* infection was widespread among goats in the study areas. To ascertain the current situation of *N. caninum* infection in goats in Thailand, however, requires a large-scale study involving a greater number of farms and animal samples from additional provinces.

## 5. Conclusions

In conclusion, it is necessary to develop accurate and effective methods to detect *N. caninum* infection in animals to aid in disease control and prevention. In this study, we constructed a synthetic NcGRA4 gene that produces a recombinant protein that may serve as an effective serological antigen to detect specific IgG in goat sera. However, the immune systems of various animal species may impact the use of recombinant protein assays for the serodetection of neosporosis in animals. Therefore, it is necessary to validate the antibody response in different animals, as well as the epitope structures of the recombinant NcGRA4.

## Figures and Tables

**Table 1:** Checkerboard assay to verify the optimal concentration of purified NcGRA4 antigen and dilution of goat sera for iELISA, represented as the mean optical density of tested positive and negative sera.

	Concentration of Purified NcGRA4 Antigen (µg/mL)	Sample Dilution	
		1:100	1:200
Positive	1	1.084	1.048
	2	1.507	1.459
Negative	1	0.343	0.299
	2	0.417	0.320

NcGRA4 *N. caninum* dense granular antigen 4, iELISA indirect enzyme-linked immunosorbent assay.

**Table 2:** Comparison of IFAT and NcGRA4 recombinant protein-based iELISAs for the detection of *N. caninum*-specific IgG antibodies.

NcGRA4- iELISA	IFAT			Sensitivity (95% CI)	Specificity (95% CI)	PPV (95% CI)	NPV (95% CI)	Kappa Value
	Positive	Negative	Total					
Positive	54	19	73	71.62	86.33	73.61	85.10	0.58
Negative	21	120	141	(59.77–81.19)	(79.22–91.36)	(61.68–82.98)	(77.9–90.33)	
Total	75	139	214					

IFAT indirect fluorescent antibody test, NcGRA4 *N. caninum* dense granular antigen 4, iELISA indirect enzyme-linked immunosorbent assay, CI Confidence interval, PPV positive predictive value, NPV negative predictive value.

## Data Availability

Data will be made available upon request.

## Supplementary Materials

The following supporting information can be downloaded at https://www.mdpi.com/article/10.3390/ani13111879/s1, Table S1: OD value of the optimal concentration of the purified NcGRA4 antigen and sample dilutions, Figure S1: Full original bolt used for Figure 3A, Figure S2: Full original bolt used for Figure 3B, Figure S3: Full original immunoblot used for Figure 4A, Figure S4: Full original immunoblots used for Figure 4B.
