# Supplementary material for: Recombinant Dense Granule Protein (NcGRA4) Is a Novel Serological Marker for Neospora caninum Infection in Goats
Source: Animals (Basel). 2023 Jun 5;13(11):1879. doi: 10.3390/ani13111879 (PMC10251823; doi:10.3390/ani13111879)
Supplement: Supplementary file 1 [file animals-13-01879-s001.zip › Data of optimal concentration of the purified NcGRA4 antigen and sample dilutions.pdf]

No	Sample	Concentration 1 µg/mL		Concentration 2 µg/mL	
		OD (1:100)	OD (1:200)	OD (1:100)	OD (1:200)
1	Negative	0.313	0.282	0.559	0.393
2	Negative	0.338	0.269	0.403	0.337
3	Negative	0.263	0.211	0.470	0.313
4	Negative	0.318	0.308	0.554	0.441
5	Negative	0.331	0.291	0.527	0.448
6	Negative	0.413	0.317	0.356	0.300
7	Negative	0.331	0.336	0.443	0.309
8	Negative	0.309	0.278	0.390	0.378
9	Negative	0.316	0.319	0.458	0.342
10	Negative	0.412	0.306	0.415	0.388
11	Negative	0.405	0.335	0.371	0.352
12	Negative	0.386	0.351	0.233	0.216
13	Negative	0.352	0.338	0.298	0.275
14	Negative	0.332	0.256	0.351	0.321
15	Negative	0.291	0.271	0.358	0.339
16	Negative	0.446	0.255	0.244	0.159
17	Negative	0.326	0.332	0.360	0.219
18	Negative	0.324	0.278	0.464	0.299
19	Negative	0.373	0.394	0.366	0.293
20	Negative	0.285	0.257	0.727	0.274
21	Positive	1.023	0.971	1.579	1.503
22	Positive	1.130	1.090	1.418	1.301
23	Positive	0.899	0.854	1.266	1.177
24	Positive	0.974	1.024	1.277	1.197
25	Positive	1.005	0.964	1.509	1.300
26	Positive	1.315	1.256	1.540	1.434
27	Positive	1.409	1.378	1.968	2.002
28	Positive	0.919	0.883	1.401	1.465
29	Positive	1.040	0.986	1.527	1.600
30	Positive	1.159	1.119	1.474	1.436
31	Positive	1.171	1.059	1.581	1.499
32	Positive	0.962	0.990	1.545	1.600
Average Negative		0.343	0.299	0.417	0.320
Average Positive		1.084	1.048	1.507	1.459
Pos/Neg Ratio		3.159	3.503	3.612	4.566
