# Supplementary material for: Recombinant Dense Granule Protein (NcGRA4) Is a Novel Serological Marker for Neospora caninum Infection in Goats
Source: Animals (Basel). 2023 Jun 5;13(11):1879. doi: 10.3390/ani13111879 (PMC10251823; doi:10.3390/ani13111879)
Supplement: Supplementary file 1 [file animals-13-01879-s001.zip › Original SDS page and immunoblot pictures.pptx]

## Slide 1
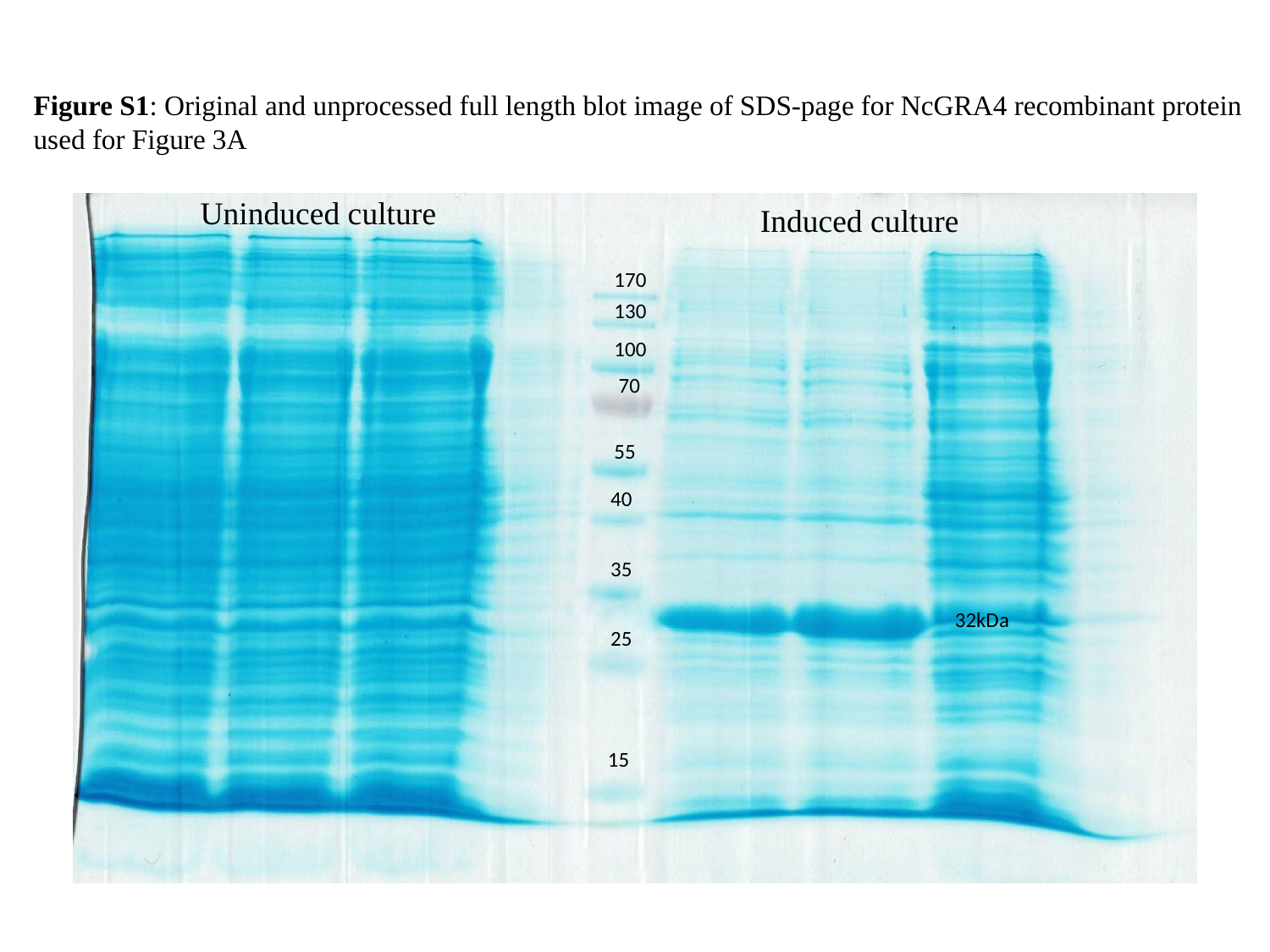

Figure S1: Original and unprocessed full length blot image of SDS-page for NcGRA4 recombinant protein used for Figure 3A
Uninduced culture
Induced culture
170
130
100
70
55
40
35
32kDa
25
 15

## Slide 2
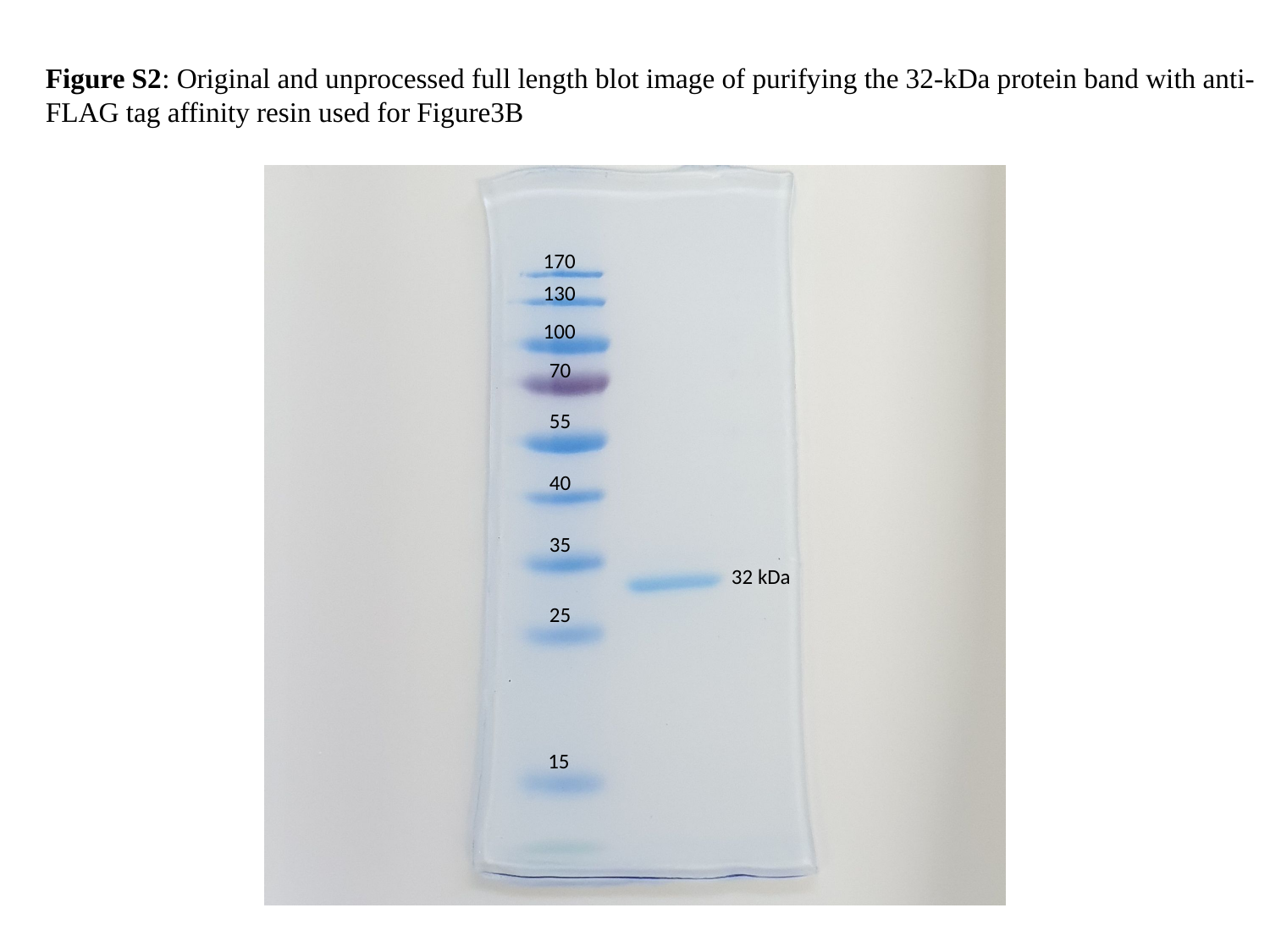

Figure S2: Original and unprocessed full length blot image of purifying the 32-kDa protein band with anti-FLAG tag affinity resin used for Figure3B
170
130
100
70
55
40
35
 32 kDa
25
 15

## Slide 3
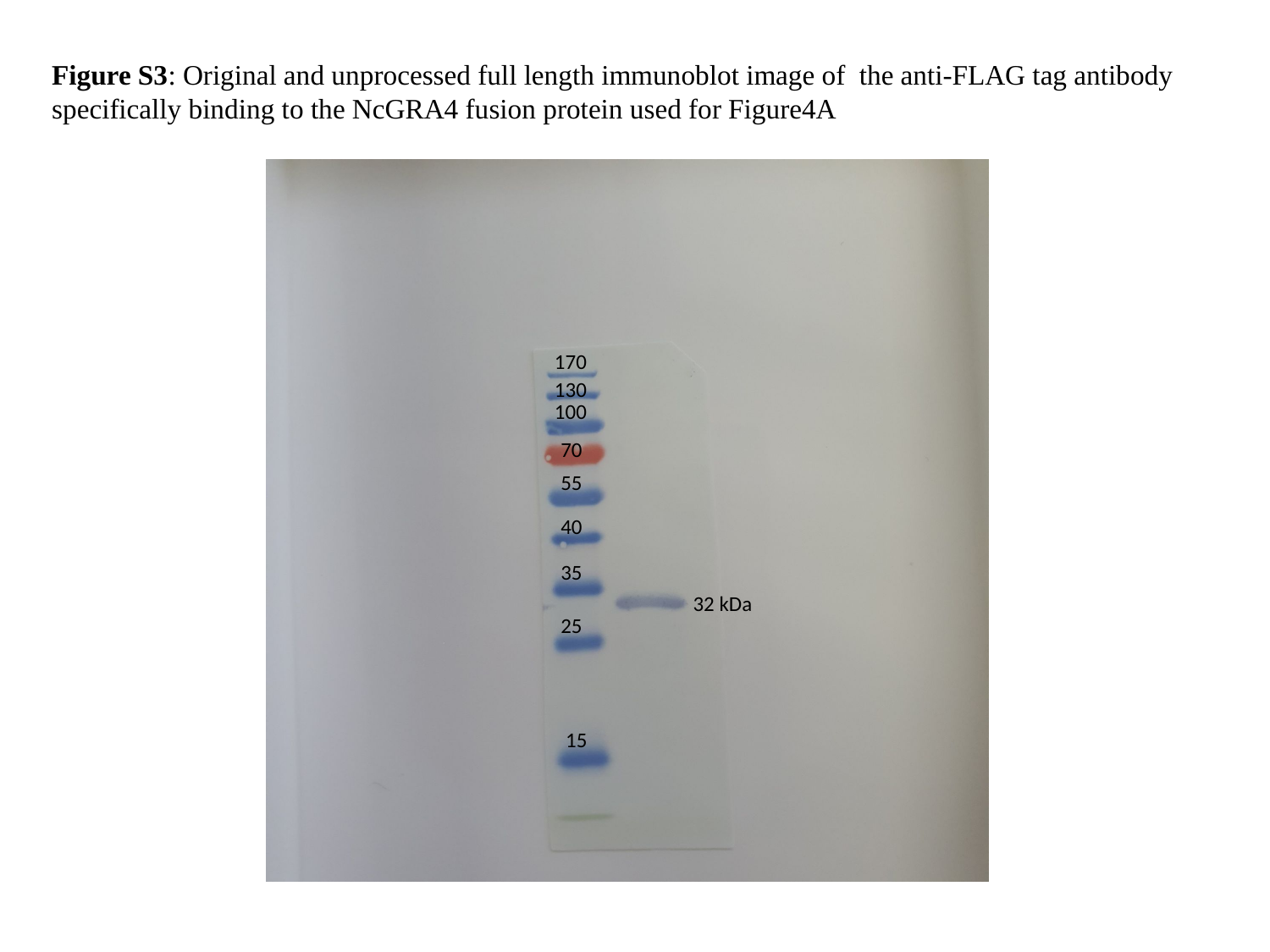

Figure S3: Original and unprocessed full length immunoblot image of the anti-FLAG tag antibody specifically binding to the NcGRA4 fusion protein used for Figure4A
170
130
100
70
55
40
35
 32 kDa
25
 15

## Slide 4
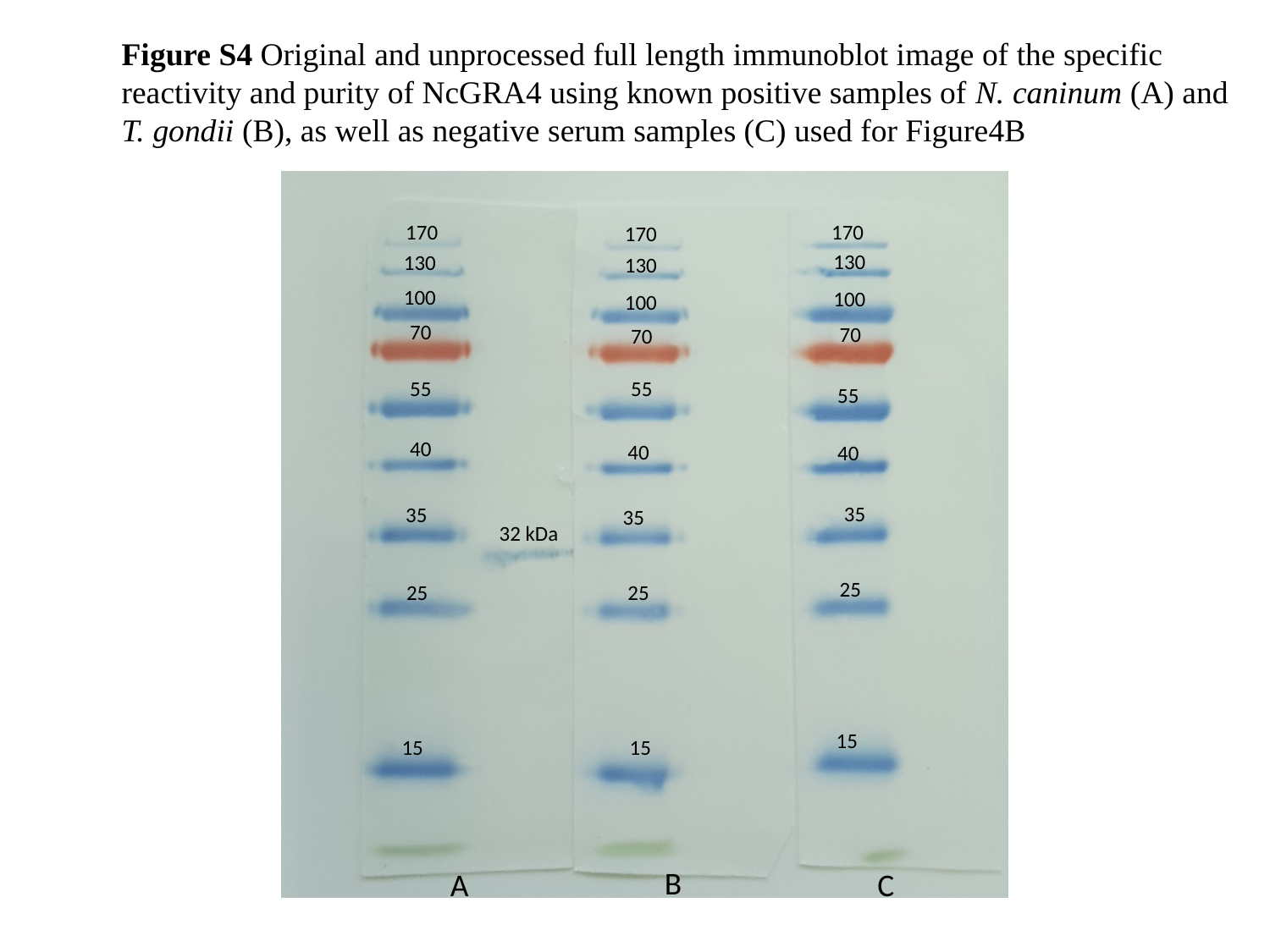

Figure S4 Original and unprocessed full length immunoblot image of the specific reactivity and purity of NcGRA4 using known positive samples of N. caninum (A) and T. gondii (B), as well as negative serum samples (C) used for Figure4B
170
170
170
130
130
130
100
100
100
70
70
70
55
55
55
40
40
40
35
35
35
 32 kDa
25
25
25
 15
 15
 15
B
A
C
